# Search term “One Health” remains of limited use to identify relevant scientific publications: Denmark as a case study

**DOI:** 10.3389/fpubh.2022.938460

**Published:** 2022-07-28

**Authors:** Guido Benedetti, Pikka Jokelainen, Steen Ethelberg

**Affiliations:** ^1^Department of Infectious Disease Epidemiology and Prevention, Statens Serum Institut, Copenhagen, Denmark; ^2^Infectious Disease Preparedness, Statens Serum Institut, Copenhagen, Denmark; ^3^Department of Public Health, Global Health Section, University of Copenhagen, Copenhagen, Denmark

**Keywords:** One Health, Denmark, literature, search term, systematic review, FAIR

## Abstract

One Health has become a popular approach, and scientific advancements in the field should be easily findable and accessible to a wide range of relevant audiences, from researchers to policymakers, and across sectors. We conducted a systematic narrative review of available scientific publications concerning One Health in the setting of Denmark that were retrievable using “One Health” as the key search term. Three searches in two databases yielded 30 retrieved publications, 13 of which were included in the review. The included publications had been published between 2015 and 2021. Twelve of the included publications were co-authored in collaboration across institutes from different sectors. Three of the included publications had focus on antimicrobial resistance, three on disease surveillance and/or control, and five were assessments or evaluations. The overall number of publications identified by a search using “One Health” as the key search term was small, and the search identified some publications that were not relevant to One Health. Our work thus highlights a missed scientific and communication opportunity of signposting articles as relevant to One Health. Using the expression “One Health” as keyword could help making One Health research more easily findable and thereby obtaining an overview of research in the field.

## Introduction

One Health has been defined as “an integrated, unifying approach that aims to sustainably balance and optimize the health of people, animals and ecosystems”, which recognizes that “the health of humans, domestic and wild animals, plants, and the wider environment (including ecosystems) are closely linked and inter-dependent” (1). The Food and Agriculture Organization of the United Nations (FAO), the World Organisation for Animal Health (WOAH), the World Health Organization (WHO) and the United Nations Environment Programme (UNEP) embrace this definition and have expressed mainstreaming One Health as their aim (1, 2).

One Health has become a popular approach globally, regionally and at country-level. In Denmark, the approach has a relatively long tradition. For example, the “Annual Report on Zoonoses in Denmark” has been published since 1994 (3), and the Danish Integrated Antimicrobial Resistance Monitoring and Research Programme (DANMAP) was established by the Danish Ministry of Food, Agriculture and Fisheries and the Danish Ministry of Health in 1995 (4). One Health institutionalization is advanced in Denmark: for example, Statens Serum Institut defines itself as a research and preparedness organization that strengthens the health of not only humans but also animals, as since 2020 it also has veterinary diagnostic and preparedness functions in partnership with University of Copenhagen (5). Denmark is an active partner in international One Health collaborations and partnerships, including the Med-Vet-Net Association (6) and the One Health European Joint Programme (7).

One Health is a challenging field due to its complexity. The outcomes from One Health work and research should be made easily findable and accessible to end-users across sectors. We conducted this review to investigate how retrievable scientific publications about One Health concerning Denmark are from scientific literature databases, by using “One Health” as the key search term. We hypothesized that we would identify publications from mid-1990s onwards, and that the majority of them would be from the most recent years, considering that “One Health” became a MeSH term in 2018 (8). Our objectives were to retrieve findable One-Health-related publications that concern Denmark, to describe the key characteristics and volume of such literature over time, and to discuss ways to improve signposting to ensure relevant audiences are reached.

## Materials and methods

We conducted a systematic narrative review of available peer-reviewed scientific publications concerning Denmark that explicitly mentioned One Health and were findable by using “One Health” as the key search term. We included no Danish equivalent for this expression because the English expression is utilized in the Danish One Health arena. To identify publications concerning Denmark, we included the name of the country and the corresponding adjective as well as names of Danish cities with a population size above 100,000, in both English and Danish, in the searches. Population, comparator, and outcome of interest were not defined.

On 29/12/2021, we searched peer-reviewed, scientific publications as available from PubMed (9) and ScienceDirect (10) using three search queries. From PubMed, we searched for (“One Health” OR “One-Health”) AND (“Denmark” OR “Danmark” OR “Danish” OR “Dansk” OR “Copenhagen” OR “København” OR “Aarhus” OR “Århus” OR “Odense” OR “Aalborg” OR “Ålborg”), limited to title and abstract. From ScienceDirect, we searched for (“One Health” OR “One-Health”) AND (“Denmark” OR “Danish” OR “Copenhagen” OR “Aarhus” OR “Odense” OR “Aalborg”), and for (“One Health” OR “One-Health”) AND (“Danmark” OR “Dansk” OR “København” OR “Århus” OR “Odense” OR “Ålborg”), both searches limited to title, abstract and author-specified keywords.

All publications that were retrieved and that were peer-reviewed scientific publications were considered as eligible for inclusion. First, we screened the retrieved titles and abstracts. Duplicates and publications that were not relevant to One Health or the Danish setting were excluded. In case of disagreement, possible reasons for eligibility, exclusion and inclusion were discussed until a shared decision was reached.

From the full-texts of the included publications, we extracted relevant available metadata. Then, we identified the institute of the affiliation of the authors and categorized them as having focus on humans, animals, food or environment. Starting in 2020, Statens Serum Institut was considered as having focus on animals in addition to humans, given the new One Health scope of the institute (5). We counted the number of cross-references and the number of cross-authorships (i.e., the same author in more than one publication) among the retrieved publications. We assumed no full namesakes. We noted the acknowledgment of funding in the publications, with a focus on references to resources explicitly dedicated for One Health or multidisciplinary collaborations.

The full-texts of the included publications were screened for the presence of the words “one health” and “one-health”. We identified the sections of the publication where the words were mentioned i.e., abstract, key words, background/introduction, materials and methods, results, discussion/conclusion and others (affiliations, acknowledgments, funding and references). The location was attributed to the discussion/conclusion category for publications without aforementioned sections. Finally, we described the topic and design of the publications, with focus on how they portrayed One Health.

The information were collated in a Microsoft Excel spreadsheet. Reporting followed the Preferred Reporting Items for Systematic Reviews and Meta-Analyses (PRISMA), as relevant and applicable to the objectives of this work (11). There was no direct contact to the authors of the retrieved and included publications. No ethical approval was required for this study.

## Results

**Figure 1:**
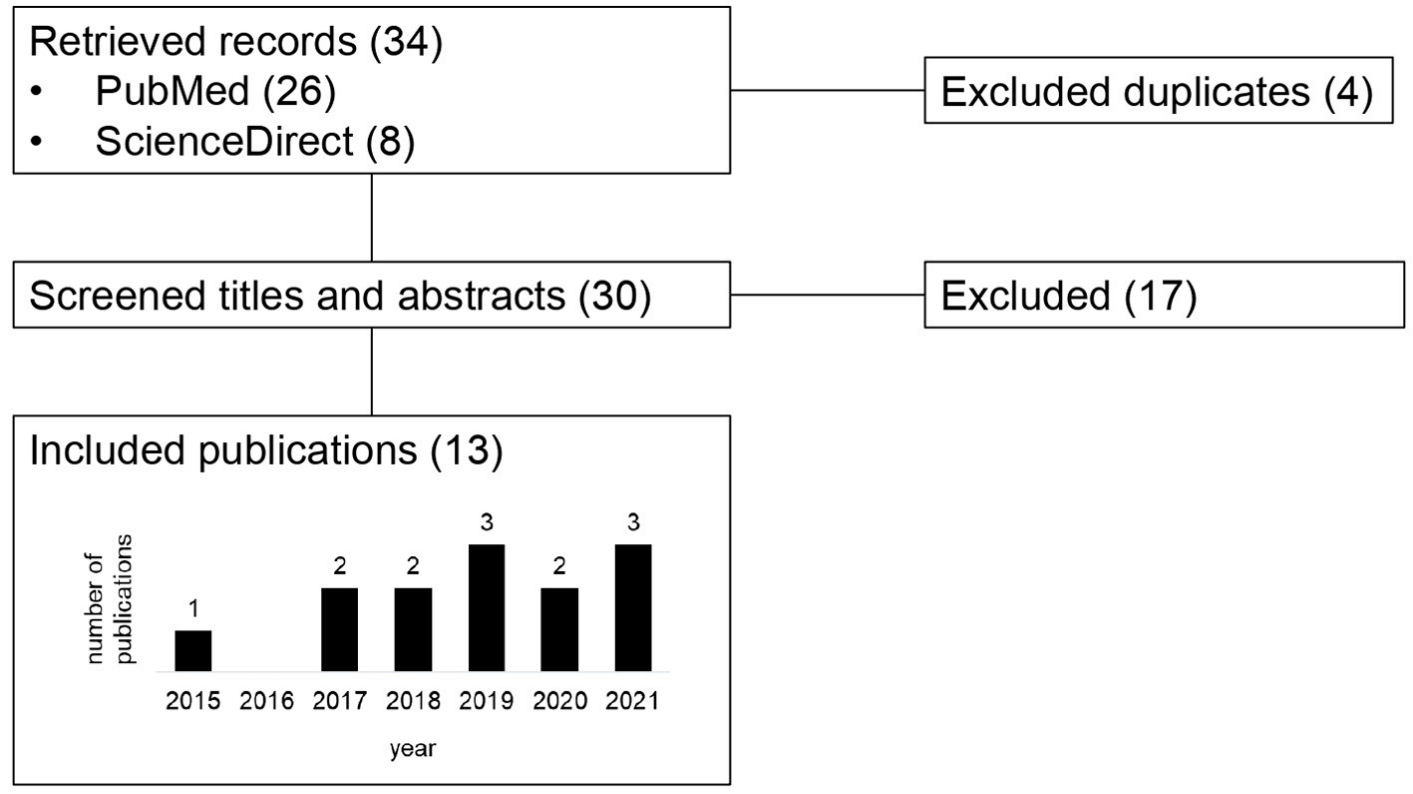
Flow diagram of retrieved, excluded and included records, by publication year.

Overall, we identified 34 entries (Supplementary Material), 26 from PubMed and eight from ScienceDirect, of which four were duplicates between the sources. The two queries in ScienceDirect produced the same eight results. All 30 retrieved publications were peer-reviewed scientific publications. Of the 30 retrieved publications, 17 were excluded. The remaining 13 publications included in the review were all written in English, and they had been published between 2015 and 2021 (Figure 1). Nine of the 13 included publications had author(s) affiliated to institutes with focus on humans, 12 to institutes with focus on animals and eight to institutes with focus on food. Twelve of the publications were co-authored in collaboration by authors from at least two institute categories.

None of the 13 publications cited each other. Five authors appeared in two of the publications. Five of the publications acknowledged funding from the European Union's Horizon 2020 Research and Innovation Programme under grant agreement No 773830: One *Health European Joint Programme*. One of the publications acknowledged funding from a grant from the University of Copenhagen to promote inter-disciplinary research. One of the publications acknowledged funding from the European Cooperation on Science and Technology (COST) Action TD 1404 Network for Evaluation of One Health.

“One Health” was in the key words of six of the 13 publications. One Health was mentioned in the background/introduction of 10 of the publications, in the results of three of the publications, and in the discussion/conclusion of nine of the publications (Table 1).

**Table 1:** Section of the publication where One Health was mentioned, among publications included in the review.

	**Abstract**	**Key words**	**Background, introduction**	**Materials and methods**	**Results**	**Discussion, conclusion**	**Affiliations, acknowledgments, funding, references**
Cordoba et al. (12)	•		•	•	NA	•	•
Alban et al. (13)		•	•				
Munk et al. (14)	•		•				
Léger et al. (15)	•	•	•	•	•	•	•
Thamsborg et al. (16)	•					•^*^	
Bjørnvad et al. (17)	•					•	•
Holmer et al. (18)	•		•			•	
Houe et al. (19)	•	•	•		•	•	•
Costagliola et al. (20)	•		•^*^			•^*^	•
Thomas-Lopez et al. (21)		•	•				•
Foddai et a. (22)	•	•	•	•		•	•
Humboldt-Dachroeden et al. (23)	•	•	•	•	•	•	•
Joensen et al. (24)							•

* *Publication without the specific sections; NA, not applicable*.

The publications included in this analysis reported various applications of One Health. Three of the 13 publications had focus on antimicrobial resistance (13, 14, 18), three on disease surveillance and/or control (19, 22, 24), one on developing One Health research projects in a clinical context (12), one on the One Health evaluation of a research center (15), one on the education of veterinary parasitology in Denmark (16), one on risk factors for obesity in dogs (17), one on outbreak investigation and management (21), one on the role of animals in the transmission of SARS-CoV-2 (20), and one on the environmental pillar of the One Health approach (23). None of the publications had evident focus on food. Five of the 13 publications were assessments or evaluations (13–15, 22, 23), three were descriptive reviews (16, 19, 20), two were observational studies (17, 18), two were descriptions of outbreaks (21, 24) and one was a study protocol (12).

In most of the publications, One Health appeared as a lens to frame the problem that was addressed or the related findings. Some publications extensively elaborated on the meaning of One Health, while others did it briefly or not at all.

## Discussion

With a simple, fast and replicable approach we reached the objectives of this review and could conclude that the peer-reviewed, scientific publications concerning Denmark scarcely mentioned “One Health” in a way that helps finding One-Health-related publications. Considering the prominence that One Health has in the Danish scientific arena, the number of retrieved publications as well as the number of included publications were small. For example, we expected to retrieve more outcomes from the substantial national investment of resources into the fight against antimicrobial resistance using One Health approach (25–27) and into international One Health programmes and networks (28, 29). Our hypothesis that we would find publications from mid-1990s onwards was not supported. The absence of retrieved publications published before 2015 was noticeable, considering the relative long history of focus on One Health at national level as well as international key milestones like the 2008 first jointly-developed tripartite guide “Zoonotic Diseases: A Guide to Establishing Collaboration between Animal and Human Health Sectors at the Country Level” (30).

Indeed, more One-Health-related scientific literature is available in the public domain. However, missing the opportunity of utilizing the term “One Health” hampers the findability of such literature—e.g., by policymakers gathering scientific evidence for One Health interventions. The results of this study can be considered to show that there is room for improvement in following the Findable, Accessible, Interoperable and Reusable (FAIR) principle for the reuse of scientific data (31, 32). Similar conclusions were recently reached also by Wind et al. (33) who recommended to utilize the term “One Health” in the title and abstract of publications about antimicrobial resistance that pertain to the One Health domain. Our observations support that recommendation, and widening it to the whole One Health domain. When One Health becomes mainstream, the need to use it as a keyword can be expected to be no longer relevant—but at present, it is useful to improve the findability of One Health research.

“One Health” has been included as a term in the Medical Subject Heading vocabulary since 2018, under the category “health” (8). This should encourage using it as a keyword. However, the current tradition of scientific literature limits the number of keywords of publications and authors might not always deem the term “One Health” relevant to be included. The journal style, editorial and peer review feedback may also affect whether and how explicitly “One Health” is mentioned in e.g., title and abstract.

The diversity of the focus areas in the included publications showcased a range of descriptions of the One Health concept, approach or perspective (34, 35). The term “One Health” appeared sometimes used as a buzz word, and it was more often found in the abstract, key words and introduction than in the methods, results and discussion of the included publications. This may suggest that the term “One Health” was included to attract readers without the concept being a key component of the work and publication (36). Moreover, the search identified some publications that were not relevant for One Health.

Translation of science into policy and then into practice is important (37), and identified barriers from science to policy in Denmark include “lack of transdisciplinary and cross-sector scientific advice” and a “lack of science for policy skills in both scientists and policymakers” (38). Better signposting of scientific articles, and using the signposting, could support more efficient science-to-policy translation.

Almost all of the included publications had authors affiliated to different sectors, demonstrating cross-sectoral collaborations. However, there was a lack of cross-referencing among the included publications. Better signposting of scientific articles could support building on previous work across research groups, and avoiding overlap and duplication of efforts. One Health networks may enable and expand the coverage and impact of One Health work (39).

Our study had several limitations. One important limitation was the unavoidable degree of subjective evaluation in categorization. Additionally, the use of only two databases of scientific literature might have reduced the number of retrieved publications. It should be emphasized that our observations and considerations are based on a limited number of very different publications, which may not represent the One Health literature well. Finally, we chose not to include other related terms such as “zoonotic” or “zoonosis”, which might be used for One Health research focusing on zoonotic pathogens and infections. Regardless, our work can serve as proof-of-concept of a simple study that produced interesting observations, which could be further investigated.

In conclusion, our study yielded an actionable set of findings to contribute to the ongoing discussions about One Health, FAIRness, cross-sectoral research collaborations and science-to-policy translation. The simple approach can be applied in similar investigations in other countries and contexts. Based on the observations from this study, we recommend more signposting to indicate that a given publication is relevant for One Health.

## Author contributions

GB and PJ together conceived the original idea, designed the study, conducted the literature search, retrieved, analyzed and interpreted the findings, and drafted the manuscript. SE contributed to the interpretation of the findings and critically revised the work. All authors approved and are accountable for all aspects of the manuscript.

## Funding

This work was supported by funding from the European Union's Horizon 2020 Research and Innovation Programme under grant agreement No. 773830: One Health European Joint Programme.

## Conflict of interest

The authors work at a governmental sector-research institute that has a One Health profile and are involved in the One Health European Joint Programme.

## Publisher's note

All claims expressed in this article are solely those of the authors and do not necessarily represent those of their affiliated organizations, or those of the publisher, the editors and the reviewers. Any product that may be evaluated in this article, or claim that may be made by its manufacturer, is not guaranteed or endorsed by the publisher.
