# Supplementary material for: Search term “One Health” remains of limited use to identify relevant scientific publications: Denmark as a case study
Source: Front Public Health. 2022 Jul 28;10:938460. doi: 10.3389/fpubh.2022.938460 (PMC9368311; doi:10.3389/fpubh.2022.938460)
Supplement: Supplementary file 1 [file Table_1.DOCX]

Supplementary Material

Retrieved, included and excluded records from PubMed – National Library of Medicine. National Center for Biotechnology Information. PubMed. Gov. (n.a.) https://pubmed.ncbi.nlm.nih.gov/ [Accessed December 29, 2021].

| Reference | Included/excluded |
| --- | --- |
| Manes C, Gollakner R, Capua I. Could Mustelids spur COVID-19 into a panzootic? Vet Ital (2020) 56(2):65-66. doi: 10.12834/VetIt.2375.13627.1. | Excluded |
| Léger A, Stärk KDC, Rushton J, Nielsen LR. A One Health Evaluation of the University of Copenhagen Research Centre for Control of Antibiotic Resistance. Front Vet Sci (2018) 5:194. doi: 10.3389/fvets.2018.00194. | Included |
| Holmer I, Salomonsen CM, Jorsal SE, Astrup LB, Jensen VF, Høg BB, et al. Antibiotic resistance in porcine pathogenic bacteria and relation to antibiotic usage. BMC Vet Res (2019) 15(1):449. doi: 10.1186/s12917-019-2162-8. | Included |
| Xia Y, Zhu Y, Li Q, Lu J. Human gut resistome can be country-specific. PeerJ (2019) 7:e6389. doi: 10.7717/peerj.6389. | Excluded |
| Houe H, Nielsen SS, Nielsen LR, Ethelberg S, Mølbak K. Opportunities for Improved Disease Surveillance and Control by Use of Integrated Data on Animal and Human Health. Front Vet Sci (2019) 6:301. doi: 10.3389/fvets.2019.00301. | Included |
| Humboldt-Dachroeden S, Mantovani A. Assessing Environmental Factors within the One Health Approach. Medicina (Kaunas) (2021) 57(3):240. doi: 10.3390/medicina57030240. | Included |
| Thamsborg SM, Johansen MV, Nejsum P, Williams AR, Mejer H. Balancing knowledge and basic principles in veterinary parasitology - Competencies for future Danish veterinary graduates. Vet Parasitol (2018) 252:117-119. doi: 10.1016/j.vetpar.2018.01.035. | Included |
| Thomas-Lopez D, Müller L, Vestergaard LS, Christoffersen M, Andersen AM, Jokelainen P, et al. Veterinary Students Have a Higher Risk of Contracting Cryptosporidiosis when Calves with High Fecal *Cryptosporidium* Loads Are Used for Fetotomy Exercises. Appl Environ Microbiol (2020) 86(19):e01250-20. doi: 10.1128/AEM.01250-20. | Included |
| Cordoba G, Sørensen TM, Holm A, Bjørnvad CR, Bjerrum L, Jessen LR. Exploring the feasibility and synergistic value of the One Health approach in clinical research: protocol for a prospective observational study of diagnostic pathways in human and canine patients with suspected urinary tract infection. Pilot Feasibility Stud (2015) 1:38. doi: 10.1186/s40814-015-0036-9. | Included |
| Bjørnvad CR, Gloor S, Johansen SS, Sandøe P, Lund TB. Neutering increases the risk of obesity in male dogs but not in bitches - A cross-sectional study of dog- and owner-related risk factors for obesity in Danish companion dogs. Prev Vet Med (2019) 170:104730. doi: 10.1016/j.prevetmed.2019.104730. | Included |
| Costagliola A, Liguori G, d'Angelo D, Costa C, Ciani F, Giordano A. Do Animals Play a Role in the Transmission of Severe Acute Respiratory Syndrome Coronavirus-2 (SARS-CoV-2)? A Commentary. Animals (Basel) (2020) 11(1):16. doi: 10.3390/ani11010016. | Included |
| Munk P, Andersen VD, de Knegt L, Jensen MS, Knudsen BE, Lukjancenko O, et al. A sampling and metagenomic sequencing-based methodology for monitoring antimicrobial resistance in swine herds. J Antimicrob Chemother (2017) 72(2):385-392. doi: 10.1093/jac/dkw415. | Included |
| Bodewes R, Zohari S, Krog JS, Hall MD, Harder TC, Bestebroer TM, et al. Spatiotemporal Analysis of the Genetic Diversity of Seal Influenza A(H10N7) Virus, Northwestern Europe. J Virol (2016) 90(9):4269-4277. doi: 10.1128/JVI.03046-15. | Excluded |
| Brabrand M, Hosbond S, Petersen DB, Skovhede A, Folkestad L. Time telling devices used in Danish health care are not synchronized. Dan Med J (2012) 59(10):A4512. | Excluded |
| Pedersen HB, Pedersen BB, Biilmann M, Møller M, Lohse N, Vedsted P, et al. Medical evacuations in Greenland in 2018: a descriptive study. Int J Circumpolar Health (2022) 81(1):2014634. doi: 10.1080/22423982.2021.2014634. | Excluded |
| Lund HH, Jessen JD. Effects of Short-Term Training of Community-Dwelling Elderly with Modular Interactive Tiles. Games Health J (2014) 3(5):277-283. doi: 10.1089/g4h.2014.0028. | Excluded |
| Alban L, Ellis-Iversen J, Andreasen M, Dahl J, Sönksen UW. Assessment of the Risk to Public Health due to Use of Antimicrobials in Pigs–An Example of Pleuromutilins in Denmark. Front Vet Sci (2017) 4:74. doi: 10.3389/fvets.2017.00074. | Included |
| Christiansen AH, Lipczak H, Knudsen JL, Kejs AMT. Risk factors for patient-reported errors during cancer follow-up: Results from a national survey in Denmark. Cancer Epidemiol (2017) 49:38-45. doi: 10.1016/j.canep.2017.05.004. | Excluded |
| Mogensen CB, Thisted AR, Olsen I. Medication problems are frequent and often serious in a Danish emergency department and may be discovered by clinical pharmacists. Dan Med J (2012) 59(11):A4532. | Excluded |
| Vandael E, Magerman K, Coenen S, Goossens H, Catry B. Antibiotic consumption in Belgian acute care hospitals: analysis of the surveillance methodology, consumption evolution 2003 to 2016 and future perspectives. Euro Surveill (2019) 24(46):1900098. doi: 10.2807/1560-7917.ES.2019.24.46.1900098. | Excluded |
| Aadahl M, Hansen BA, Kirkegaard P, Groenvold M. Fatigue and physical function after orthotopic liver transplantation. Liver Transpl (2002) 8(3):251-9. doi: 10.1053/jlts.2002.31743. | Excluded |
| Joensen KG, Schjørring S, Gantzhorn MR, Vester CT, Nielsen HL, Engberg JH, et al. Whole genome sequencing data used for surveillance of *Campylobacter* infections: detection of a large continuous outbreak, Denmark, 2019. Euro Surveill (2021) 26(22):2001396. doi: 10.2807/1560-7917.ES.2021.26.22.2001396. | Included |
| Jones RW, McCrone P, Guilhaume C. Cost effectiveness of memantine in Alzheimer's disease: an analysis based on a probabilistic Markov model from a UK perspective. Drugs Aging (2004) 21(9):607-20. doi: 10.2165/00002512-200421090-00005. | Excluded |
| Cornago D, Garattini L. The stoma appliances market in five European countries: a comparative analysis. Appl Health Econ Health Policy (2002) 1(1):43-50. | Excluded |
| Bodewes R, Rubio García A, Brasseur SM, Sanchez Conteras GJ, van de Bildt MW, Koopmans MP, et al. Seroprevalence of Antibodies against Seal Influenza A(H10N7) Virus in Harbor Seals and Gray Seals from the Netherlands. PLoS One (2015) 10(12):e0144899. doi: 10.1371/journal.pone.0144899. | Excluded |
| Johannessen JO, McGlashan TH, Larsen TK, Horneland M, Joa I, Mardal S, et al. Early detection strategies for untreated first-episode psychosis. Schizophr Res (2001) 51(1):39-46. doi: 10.1016/s0920-9964(01)00237-7. | Excluded |

Retrieved, included and excluded records from ScienceDirect – Elsevier’s ScienceDirect, Advanced Search (n.a.). https://www.sciencedirect.com/search [Accessed December 29, 2021].

| Reference | Included/excluded |
| --- | --- |
| Foddai A, Nauta M, Ellis-Iversen J. Risk-based control of *Campylobacter* spp. in broiler farms and slaughtered flocks to mitigate risk of human campylobacteriosis – A One Health approach. Microbial Risk Analysis (2021) 100190. https://doi.org/10.1016/j.mran.2021.100190. | Included |
| Thamsborg SM, Johansen MV, Nejsum P, Williams AR, Mejer H. Balancing knowledge and basic principles in veterinary parasitology - Competencies for future Danish veterinary graduates. Vet Parasitol (2018) 252:117-119. doi: 10.1016/j.vetpar.2018.01.035. | Included * |
| van Deurzen I, Rod NH, Christensen U, Hansen ÅM, Lund R, Dich N. Neighborhood perceptions and allostatic load: Evidence from Denmark. Health Place (2016) 40:1-8. doi: 10.1016/j.healthplace.2016.04.010. | Excluded |
| Aadahl M, Hansen BA, Kirkegaard P, Groenvold M. Fatigue and physical function after orthotopic liver transplantation. Liver Transpl (2002) 8(3):251-9. doi: 10.1053/jlts.2002.31743. | Excluded * |
| Bjørnvad CR, Gloor S, Johansen SS, Sandøe P, Lund TB. Neutering increases the risk of obesity in male dogs but not in bitches - A cross-sectional study of dog- and owner-related risk factors for obesity in Danish companion dogs. Prev Vet Med (2019) 170:104730. doi: 10.1016/j.prevetmed.2019.104730. | Included * |
| Johannessen JO, McGlashan TH, Larsen TK, Horneland M, Joa I, Mardal S, et al. Early detection strategies for untreated first-episode psychosis. Schizophrenia Research (2001) 51;1:39-46. https://doi.org/10.1016/S0920-9964(01)00237-7. | Excluded |
| Christiansen AH, Lipczak H, Knudsen JL, Kejs AMT. Risk factors for patient-reported errors during cancer follow-up: Results from a national survey in Denmark. Cancer Epidemiol (2017) 49:38-45. doi: 10.1016/j.canep.2017.05.004. | Excluded * |
| Skov T, Cordtz T, Kirkeskov Jensen L, Saugman P, Schmidt K, Theilade P. Modifications of health behaviour in response to air pollution notifications in Copenhagen. Social Science & Medicine (1991) 33;5:621-626. https://doi.org/10.1016/0277-9536(91)90220-7. | Excluded |

* duplicate, also retrieved from PubMed
